# Evaluation of aquaporins in the cerebrospinal fluid in patients with idiopathic normal pressure hydrocephalus

**DOI:** 10.1371/journal.pone.0258165

**Published:** 2021-10-01

**Authors:** Laura Hiraldo-González, José Luis Trillo-Contreras, Pablo García-Miranda, Rocío Pineda-Sánchez, Reposo Ramírez-Lorca, Silvia Rodrigo-Herrero, Magdalena Olivares Blanco, María Oliver, Maria Bernal, Emilio Franco-Macías, Javier Villadiego, Miriam Echevarría

**Affiliations:** 1 Institute of Biomedicine of Seville (IBiS), Virgen del Rocío University Hospital, (HUVR)/Spanish National Research Council (CSIC)/University of Seville, Seville, Spain; 2 Department of Physiology and Biophysics, University of Seville, Seville, Spain; 3 Clinical Neuroscience Management Unit, Neurology Service, University Hospital Virgen del Rocío, Seville, Spain; 4 Clinical Neuroscience Management Unit, Neurosurgery Service, University Hospital Virgen del Rocío, Seville, Spain; 5 Network Center for Biomedical Research in Neurodegenerative Diseases (CIBERNED), Seville, Spain; Goethe University Hospital Frankfurt, GERMANY

## Abstract

Brain aquaporin 1 (AQP1) and AQP4 are involved in cerebrospinal fluid (CSF) homeostasis and might participate in the origin of hydrocephalus. Studies have shown alterations of perivascular AQP4 expression in idiopathic normal pressure hydrocephalus (iNPH) and Alzheimer’s disease (AD). Due to the overlapping of clinical signs between iNPH and certain neurological conditions, mainly AD, specific biomarkers might improve the diagnostic accuracy for iNPH. The goal of the present study was to analyze and quantify the presence of AQP1 and AQP4 in the CSF of patients with iNPH and AD to determine whether these proteins can be used as biomarkers of iNPH. We examined AQP1 and AQP4 protein levels in the CSF of 179 participants (88 women) classified into 5 groups: possible iNPH (81 participants), hydrocephalus associated with other neurological disorders (13 participants), AD (41 participants), non-AD dementia (32 participants) and healthy controls (12 participants). We recorded each participant’s demographic and clinical variables and indicated, when available in the clinical history, the record of cardiovascular and respiratory complications. An ELISA showed virtually no AQP content in the CSF. Information on the vascular risk factors (available for 61 patients) confirmed some type of vascular risk factor in 86% of the patients with possible iNPH and 58% of the patients with AD. In conclusion, the ELISA analysis showed insufficient sensitivity to detect the presence of AQP1 and AQP4 in CSF, ruling out the possible use of these proteins as biomarkers for diagnosing iNPH.

## Introduction

Idiopathic normal pressure hydrocephalus (iNPH) is a disease commonly observed in older adults and is generally underdiagnosed because the obvious symptoms are frequently interpreted as indicative of the elderly condition. The prevalence in the general population is still unclear but figures ranging from 0.5% to 2.9% are indicated among those aged 65 and older [1].

The typical clinical triad of this potentially reversible neurological condition consists of gait disturbance, dementia and urinary incontinence, combined with ventriculomegaly [2–4]. Although ventricular enlargement is not a symptom particular to iNPH, given that this neuroradiological sign can be observed in various neurodegenerative and vascular conditions, the origin of the enlargement is still controversial, and mechanical, vascular, inflammatory and metabolic factors have been indicated [4]. For instance, iNPH and Alzheimer’s disease (AD) often coexist [5–7], and can sometimes be confused because iNPH can present the canonical symptoms of AD, such as extensive memory loss and executive dysfunction [6]. However, given the relative reversibility of iNPH after lumbar drainage of CSF volume with significant improvement of the patient’s condition, an early and differential diagnosis of the two pathological conditions would be highly desirable. For instances several studies have examined in CSF the presence of biomarkers typically used for diagnosis of Alzheimer disease as amyloid β (Aβ), Tau and P-Tau [6,7], looking for a possible use to separate patients with iNPH from patients with other neurodegenerative disorders.

Brain aquaporins (AQPs), particularly AQP4 and AQP1, are water channel proteins that facilitate the flow of water through the brain compartments and play an important role in CSF homeostasis [8,9]. Recent findings have revealed that the glymphatic system facilitates fluid and waste clearance from the brain through a mechanism that depends on the presence of AQP4 in the perivascular astroglia [10,11]. Loss or mislocalization of astrocyte AQP4 and perivascular reactive astrogliosis have been observed in animal and human studies of iNPH and AD [12–14]. Experiments using aged animals exposed to hypoxia have demonstrated the development of a condition that recalls parameters observed in patients with iNPH, such as ventricular enlargement and impaired cognitive function, with clear participation of AQP4 [8]. Glymphatic transport was suppressed in a mouse model (APP/PS1) of AD, a reduction that occurs prior to the significant accumulation of amyloid-beta [15]. An electron microscopy analysis of cortical brain biopsies from patients with iNPH demonstrated a significant reduction in AQP4 density in astrocyte endfoot membranes along microvessels when compared with controls [13,16]. Additionaly, decreased gadobutrol clearance from the subarachnoid space, indicative of reduced glymphatic clearance in iNPH has been observed and postulated that reduced glymphatic function is instrumental for dementia in this disease [17].

The obvious participation of AQP4 in CSF transport in the brain and interstitial fluid clearance as a key element of the glymphatic system, as well as the lack of reliable biological markers in the differential diagnosis of iNPH and AD, lead to the conclusion that brain AQPs might be involved in the development of these pathological conditions and might help monitor and differentiate the two diseases. Thus, the aim of this study is to determine whether ventricular enlargement in elderly patients with iNPH and patients with AD might correlate with abnormal expression of either AQP1 or AQP4 in the brain, affecting the abundance of these proteins in CSF.

## Materials and methods

This observational, cross-sectional, and retrospective study was designed to analyze the expression of AQPs on CSF samples of patients with “probable iNPH” against patients with a different diagnosis, and healthy controls. The study was conducted according to the guidelines of the Declaration of Helsinki, and approved by the Ethics Committee of University Hospital Virgen Macarena and University Hospital Virgen del Rocío (Protocol Version: 1; date of approval 21/12/2016).

### Patient characteristics and CSF collection

CSF samples were donated, after signing informed consent, by 179 patients (88 female) aged over 50 years old. They were recruited from December 2018 until January 2020 from Virgen del Rocío University Hospital, Seville, Spain. For diagnosis, patients were classified into 5 groups (Table 1):

I- Groups 1–2 (possible iNPH and unlikely iNPH). 94 patients were recruited from the Department of Neurosurgery and Neurology. These patients presented with gait disorder (with or without cognitive impairment or urinary incontinence) and cranial magnetic resonance imaging (MRI) showing free communication between the ventricular system and subarachnoid space and an Evans’ index >0.30. They were evaluated by expert neurologists considering the diagnosis of iNPH. The workup included Hellström’s scale [18]; cranial MRI re-evaluation considering the presence of a disproportionately enlarged subarachnoid-space hydrocephalus (DESH) pattern [19], CSF tap test (CSF TT) (positive if improvement was documented on Hellström’s scale), and lumbar infusion test with a calculation of the resistance to CSF outflow (Rout) (positive if Rout >12 mm Hg/min/ml Rout) [20]. Group 1 was made up by 81 out of 94 patients with “possible iNPH” according to the following criteria consistent with international guidelines [1,21,22]: 1) only symptoms of Hakim’s triad; 2) DESH pattern observed on cranial MRI; 3) a positive CSF TT or lumbar infusion test; and 4) improvement after applying a shunt. On the contrary, Group 2 was made up by the remaining 13 patients diagnosed with “unlikely iNPH” according to atypical symptoms, not consistent DESH pattern, negative CSF TT, and negative lumbar infusion test. In consequence, a placement of the shunt was not here indicated.
II- Groups 3–4 (AD suspected). Both groups were recruited from the Neurology Department and included patients with cognitive complaints in whom AD was suspected after a comprehensive clinical, neuropsychological, and radiological evaluation. Group 3 was made up by 41 patients who met the International Working Group (IWG) 2 criteria [23] for typical or atypical AD with decreased Aβ1–42 coupled with increased total tau or phosphorylated tau in CSF. On the contrary, Group 4 was constituted by 32 patients with cognitive impairment who were negative for CSF AD biomarkers and did not show hydrocephalus on MRI.
III. Group 5 (healthy controls). This group was recruited by Department of Anesthesia and made up by 12 not-cognitively-impaired patients for whom a CSF sample was obtained during spinal anesthesia for non-neurological surgical indications.

**Table 1:** Demographic and clinical variables of 179 patients included in the CSF-AQPs analysis.

Diagnosis	Number of Patients	Sex Female/Male	Mean Age at Inclusion, years ± SD (range)	AQP1^+^ Samples (Positive/Total)	AQP4^+^ Samples (Positive/Total)
1. Possible iNPH	81	36/45	74.53 ± 5.67 (60–86)	6/17	0/81
2. Hydrocephalus associated with other neurological disorders	13	4/9	71.69 ± 7.33 (54–80)	0/5	0/13
3. AD	41	23/18	70.78 ± 5.1 (56–79)	3/10	3/41
4. Non-AD dementia	32	15/17	68.00 ± 7.4 (48–79)	ND	2/32
5. Healthy Controls	12	10/2	61.05 ± 8.75 (45–70)	2/6	2/12

AD, Alzheimer’s disease; ND, not determined.

For the analysis, demographic and clinical variables were recorded for each participant, including gender, age at study inclusion, time from symptoms onset, and clinical diagnosis (Table 1). CSF samples were always obtained by lumbar puncture done in the intervertebral region L3/L4 or L4/L5 during the removal of CSF at the tap test, just after measuring pressure, when iNPH was suspected (Groups 1–2); as a procedure for diagnosing AD by CSF biomarkers (Groups 3–4), or during spinal anesthesia (Group 5). We calculated that when the suspicion is iNPH, the average from the onset of symptoms to our consultation was 3 years; and when the suspicion is Alzheimer’s it was somewhat less, among 1–2 years.

After extraction, CSF was aliquoted and kept at -80°C until use in the Biobank of the University Hospital Virgen del Rocío, Seville, Spain.

### Transfection with AQP4

HEK293T-plated cells at approximately 80% confluence were transfected with AQP4-EGFP construct using Lipofectamine 2000 (Invitrogen) as described by Sánchez-Gomar et al. [24]. Thirty-two hours later, the cells were harvested and lysed in radioimmunoprecipitation assay buffer (10 mM Tris-HCl pH 7.5, 150 mM NaCl, 1 mM EDTA, 0.1% SDS, 1% Triton X-100) with protease inhibitors. Protein levels were measured by the Bradford method using gamma globulin as the standard. Different concentrations of the homogenate were used as a positive control for AQP4 enzyme-linked immunosorbent assay (ELISA).

### AQP ELISA

For the AQP CSF determination, CSF was collected into polypropylene tubes and stored at -80°C until analysis by ELISA. Quantification of the AQP1 and AQP4 protein content in 100 μL of CSF from patients and healthy controls was performed using commercial ELISA kits for AQP1 and for AQP4 (SEA579Hu and SEA582Hu, respectively; Cloud-Clone Corp), following the manufacturer’s protocol. The optical density obtained in the ELISA plates was measured with a spectrophotometer at 450 nm (Thermo Scientific). For the standard curve, we used lyophilized protein of either AQP1 or AQP4 provided with the kit. Homogenates of the protein extracts were prepared for use as positive controls for the presence of AQP4. Bloody CSF was also included in the analysis as a positive control for AQP1 detection, given that AQP1 is highly abundant in human erythrocytes [25].

### Statistical analysis

The specific number of CSF analyzed in each experimental condition (n) are exposed in each figure. Data are presented as mean ± standard deviation (SD). For all the statistical analysis performed, data were tested for normality (Kolmogorov-Smirnov, Shapiro-Wilk, D’Agostino & Pearson and Anderson-Darlin tests) and equal variance (Brown-Forsythe test). Because the data do not adjust to a normal distribution, the non-parametric Kruskall-Wallis test was carried out. All statistical analysis were conducted by GraphPad Prism8 software.

## Results and discussion

A combination of various tests that includes a neurological examination, neuroimaging study and tap test are usually performed to diagnose iNPH [26,27]; however, the tests are often unable to differentiate iNPH from other neurobehavioral disorders that progress with memory problems or attention deficits, as exhibited by patients with AD. A specific biomarker for iNPH is therefore required to improve diagnostic accuracy. AQP proteins in human CSF have been previously analyzed to find biomarkers for certain neurological diseases [28–31]. Given that recent findings have revealed AQP1 and AQP4 as key players in CSF homeostasis and that disturbance of their expression in the brain is an indication of the onset of iNPH [9,13], in the present study we evaluated the presence of these proteins in the CSF of patients with this disease (Table 1). In addition to controls, we recruited patients with AD for comparison, given the overlapping symptoms between some patients with AD and those diagnosed with possible or questionable iNPH (Table 1).

We used two specific ELISA kits to determine the presence of AQP1 and AQP4 in CSF [28–32]. The results demonstrated that neither of these two proteins were detectable at significant levels in most of the analyzed CSF samples (Table 1). Given that the presence of AQP1 and AQP4 in human CSF has previously been indicated by a number of authors [29,33], we performed additional experimental checks to determine the confidence and reinforce our somewhat unexpected negative findings.

**Fig 1:**
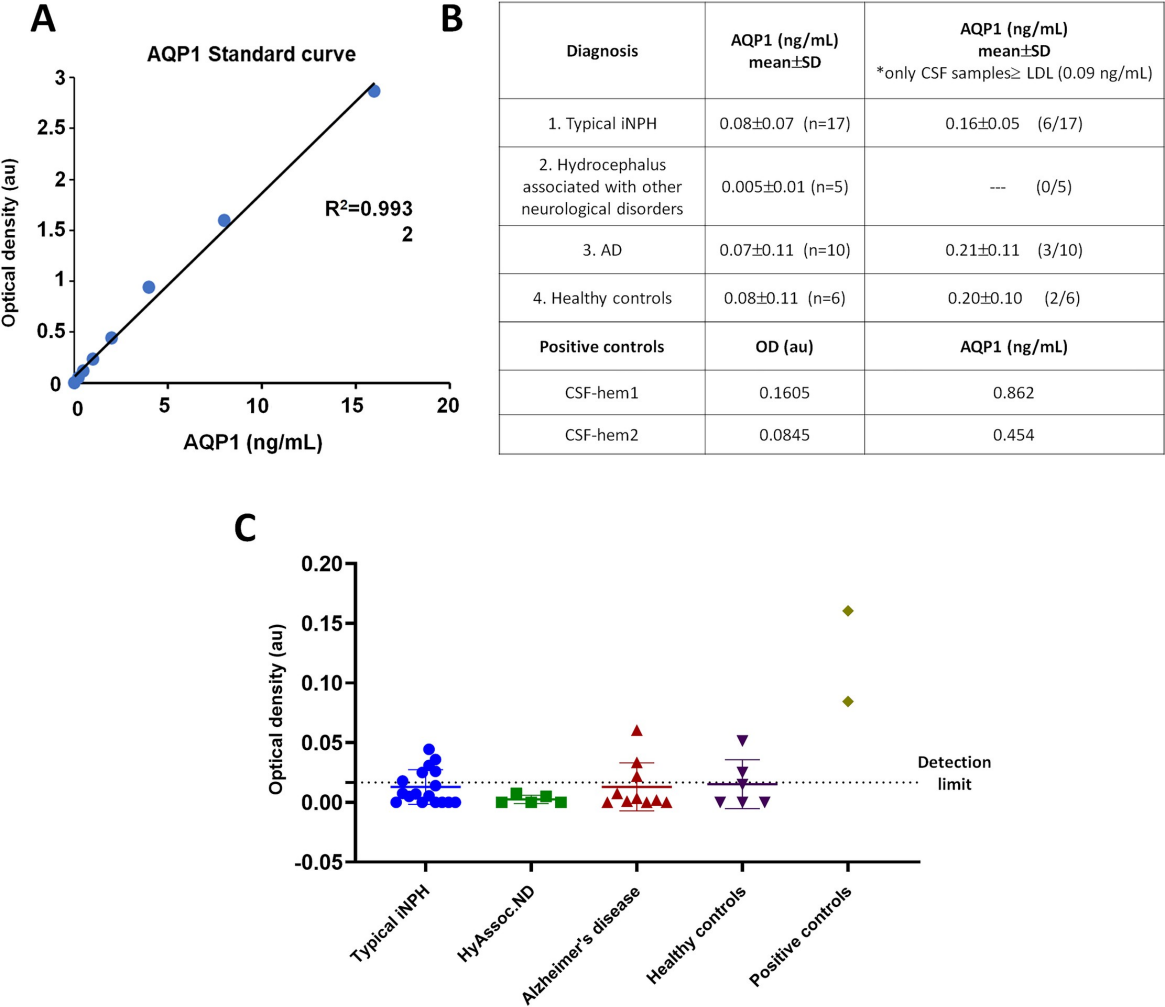
AQP1 detection by ELISA. The graph represents the standard curve obtained with the ELISA kit (A). The table indicates the type of sample, the mean AQP1 concentration per group including all analyzed samples (central column) and the mean AQP1 concentration when only samples with levels above the lower detection level (LDL) were considered (right column). The two lower rows show the optical density and amount of AQP1 calculated for the bloody samples (hem1 and hem2) (B). Representation of AQP1-OD for each individual patient divided according to the clinical diagnosis, in which the dot line plot indicates the lower detection level of the commercial kit (C). Non statistical differences were observed among the different groups.

Two CSF samples from patients with hemorrhagic hydrocephalus who were initially excluded were subsequently included in a second round of assays. The inclusion of these two CSF samples, which were slightly contaminated by erythrocytes that abundantly express AQP1 in their plasma membrane, provide us with a perfect positive control for the ELISA test and demonstrate that, if AQP1 was present, we would be able to detect it in our analysis. As shown in Fig 1, the standard curve prepared by serial dilutions from a stock solution of 16 ng/mL of AQP1 protein (provided by the kit) generated a sharp regression line (R^2^ = 0.9932) that ensured the exact determination of AQP1 protein in the detection range covered by the standard curve. As shown in Fig 1B, the absorbance values were significantly high (as to be interpolated in the standard curve) only in the two CSF samples with traces of blood (CSF-hem1 and CSF-hem2), allowing us to determine the presence of AQP1 in those samples (Fig 1B). The absorbance values for the rest of the analyzed CSF samples were extremely low, almost zero (Fig 1C).

Therefore, the estimated AQP1 concentration was above the lower detection limit (LDL) of 0.09 ng/mL in only a few CSF samples from the various patient groups (Fig 1B). In most of the tested CSF samples, the AQP1 concentrations did not reach the lower level of the sensitivity range in the standard curve created for the calculations, thereby revealing the absence of AQP1, at least in the range of concentrations (0.25 to 16 ng/mL) susceptible for determination by the ELISA kit (Table 1, Fig 1B).

**Fig 2:**
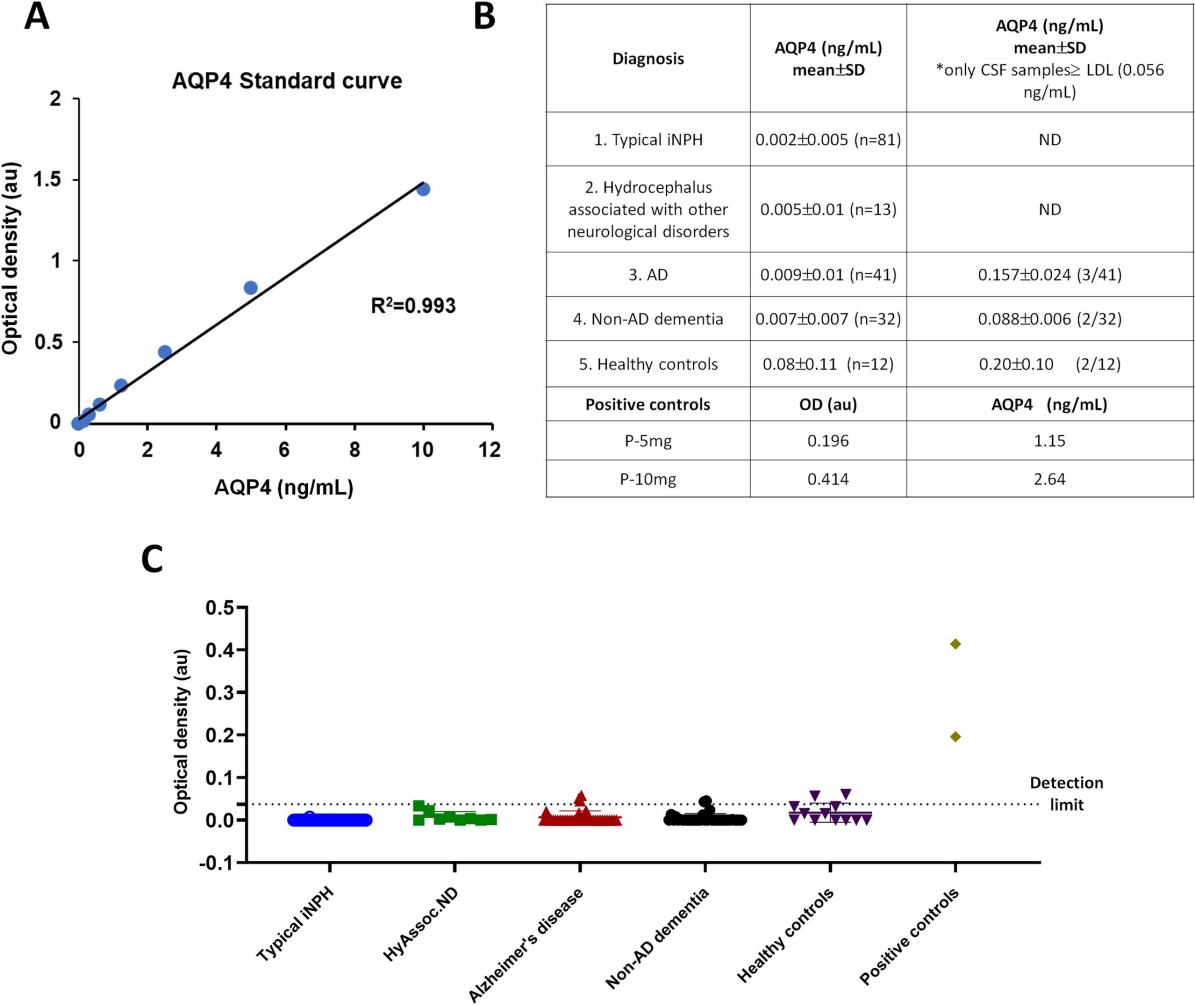
AQP4 detection by ELISA. The graph represents the standard curve obtained with the ELISA kit (A). The table indicates the type of sample, the mean AQP4 concentration per group including all analyzed samples (central column), the mean AQP4 concentration when only the samples with levels above the lower detection level (LDL) were considered (right column). The two lower rows show the optical density (OD) value and the amount of AQP4 for the positive control samples (P-5mg and P-10mg) (B). Representation of AQP4-OD for each individual patient divided according to the clinical diagnosis, in which the dot line plot indicates the lower detection level of the commercial kit (C). Non statistical differences were observed among the different groups.

Regarding AQP4 detection in CSF, given that AQP4 is not expressed in the erythrocyte membrane, we generated a specific positive control for this assay to further demonstrate the adequacy of the commercial ELISA test for detecting AQP4 proteins. HEK cells were transfected with a plasmid encoding for human AQP4, and a homogenate of proteins extracted from these cells was prepared at different concentrations and used in a trial test of the ELISA assay. In the case of AQP4, the standard curve covered a determination range from 0.156 ng/mL to 10 ng/mL (Fig 2A); and the high coefficient of the regression line ensures the precise determination of AQP4 protein within the given range. As shown in Fig 2B, the presence of AQP4 protein was detected in the homogenate of transfected HEK cells and in only a few AD samples (3 samples out of a total of 41), non-AD dementia samples (2/32) and healthy control samples (2/12) at very small concentrations. However, AQP4 was not detected in any CSF sample from the patients with iNPH or other hydrocephalic condition associated with other neurological disorders, thereby confirming the absence of AQP4 in the CSF of most of the patients analyzed in the present study. The optical density values were always very low when measuring the patients’ CSF but increased considerably when we used samples from the homogenate of AQP4-transfected HEK cells (5 or 50 mg), as can be observed in Fig 2C.

In 2011, Blocher et al. [28] were the first to report the presence of AQP1 and AQP4 in human CSF, namely in patients with bacterial meningitis. However, the authors did not rule out the possibility that the significant increase in AQP1 concentrations was due to higher expression and subsequent shedding into the CSF or due to possible cell damage induced by the bacterial infection. AQP1 levels in CSF have also been evaluated in 16 full-term infants with congenital hydrocephalus, detecting significant increases in AQP1 in obstructive cases when compared with patients with communicating hydrocephalus and controls [33]. The authors theorized that the increase in CSF AQP1 content in obstructive hydrocephalus might be due to a compensatory mechanism whereby AQP1 leaking into the CSF would reduce CSF production, thereby ameliorating the high intracranial pressure built up in the obstructive condition.

Two previous studies [29,34] explored AQP levels in the CSF of patients with iNPH. One study compared levels of AQP1 and tumor necrosis factor alpha in healthy older adult controls, patients with mild cognitive impairment and patients with iNPH [34]. The other study employed AQP4 levels and biomarkers for diagnosing AD (amyloid-β, total tau and phosphorylated tau), were analyzed in CSF of iNPH and patients with AD. In the first study, the results showed lower protein content in the CSF of patients with iNPH compared with the controls, and the absolute AQP1 values (in ng/mL) in the CSF of patients with iNPH were lower than those of the other two groups. The second study [29] reported a reduction in AQP4 levels in the CSF of patients with iNPH, levels that were even lower in the CSF of patients with AD, when compared with the control group. Previous reports have therefore demonstrated a reduction in the specific AQP analyzed in the CSF of patients with iNPH and do not support a diagnostic value for AQP1 or AQP4 content for iNPH, similar findings to those of our study.

The reason behind the almost complete lack of these proteins in the CSF of the patients with iNPH and not just a reduction in AQP levels as indicated by other authors remains unclear. Differences in the kits’ sensitivity could offer one explanation, but that was not the case for our study. Inadequate criteria for extrapolating the protein concentrations might have been the cause, as observed in the case of AQP4. Specifically, the detection range for the ELISA kit used in the study by Arighi et al. was 0.156 ng/mL to 10 ng/ mL [29], indicating that the assay’s sensitivity (or LDL), defined as the lowest protein concentration that could be differentiated from zero, was 156 pg/mL. The mean AQP4 value of 1.07 pg/mL in the CSF of patients with iNPH reported by Arighi et al. [29] is more than 100 times lower than the kit’s LDL. Such small values should be taken as below the acceptable range for accurate estimation, and therefore the protein concentration cannot be differentiated from zero, as we reported in the present study.

As in AD, the loss of perivascular AQP4 in iNPH has been observed by immunohistochemical analysis [12,13,35], which could explain the reduction or nonexistence of AQP4 in the patients’ CSF. The striking absence of detectable amounts of AQP1 and AQP4 in CSF defeats any hope of considering AQP proteins in the CSF as potential biomarkers for classifying and differentiating iNPH and AD. More sensitive techniques for detecting these proteins at lower concentrations in CSF could offer some hope for their future use as biomarkers of these neurological diseases. In that sense presence of aquaporin-4 microparticles in CSF of patients with NMO have been found and proposed as possible tool to be used for early diagnostic purposes [36]. The relationship between AQPs and the potential origin of these neurological disorders is not, however, brought in question by these negative results [37].

Although iNPH is generally considered an idiopathic condition, its etiology appears to combine several factors. Abnormal CSF dynamics, resulting from alterations in CSF pulsation due to variations in cardiac rhythm, respiration rate, or brain compliance, can play a role [38]. The association with diabetes mellitus and vascular etiology have also been indicated [39]; and sleep-disorder breathing as observed in obstructive sleep apnea has been indicated to affect the proper circulation of interstitial CSF into the glymphatic circulation contributing to cause iNPH [40].

We observed the presence of cardiovascular and respiratory risk factors that could contribute to iNPH in the clinical history of a subset of patients (Table 2). Interestingly, the general analysis of the clinical data indicated a clear association between cardiovascular risk factors and, to a lesser proportion, respiratory diseases (Table 2), as indicated in previous studies [38,39,41], thereby suggesting that these factors could contribute to the pathophysiology of iNPH and AD. Cardiovascular disorders and diabetes can contribute to the impairment of the glymphatic system [16], and alterations in AQP expression in these scenarios have been indicated.

**Table 2:** Demographic and clinical variables of 73 patients with an associated pathological factor other than neurological disease.

Diagnosis	Number of Patients	Sex (Female/Male)	Mean Age at Inclusion, years ± SD (range)	Vascular Risk Factors, n (%)	Respiratory Diseases, n (%)
1. Possible iNPH	36	16/20	73.30 ± 5.50 (57–84)	32 (89%)	4 (11%)
2. Hydrocephalus associated with other neurological disorder	13	4/9	71.69 ± 7.33 (54–80)	10 (77%)	2 (15%)
3. AD	12	6/6	63.08 ± 8.09 (52–76)	7 (58%)	1 (8%)
4. Healthy controls	12	10/2	61.05 ± 8.75 (45–70)	ND	ND

Respiratory diseases include chronic obstructive pulmonary disease, asthma and obstructive sleep apnea syndrome. AD, Alzheimer’s disease; ND,: Not determined.

Finally, we would like also to say that this study is not free of limitations. For instances, none of the different criteria used for diagnosis show high enough sensitivity and specificity for diagnosing iNPH, so some overlap between groups 1–2 could be present. Also, the apparently clear separation between groups 1 and 3 has recently been challenged by a new line of evidence that supports a continuum between AD and iNPH [7]. But in absence of specific biomarkers for iNPH, we followed recommendations from clinical practice guidelines for making the best possible discrimination among groups. For all these reasons we should emphasize on that a specific biomarker for iNPH is therefore required to improve diagnostic accuracy.

In summary, AQPs have been widely implicated in CSF homeostasis. AQP1 in the choroid plexus and AQP4 in brain perivascular blood vessels play important roles in CSF production and clearance. Alterations in the glymphatic system associated with the reduction or redistribution of AQP4 expression appear to participate in the etiology of iNPH and AD. However, the shedding of AQP1 and AQP4 into the CSF does not appear to occur in any of these diseases, at least not at levels detectable by ELISA. Thus, detecting AQPs in CSF as biomarkers for diagnosing iNPH and distinguishing these patients from those with AD appears unlikely, at least with the detection level offered by the currently available commercial ELISA kits.

## Conclusions

AQP1 and AQP4 proteins are almost undetectable in the CSF of patients with iNPH using commercial ELISA tests. These AQPs were also undetectable or rarely present in the CSF of patients with AD and healthy controls. AQP1 and AQP4 cannot be used as CSF biomarkers to distinguish iNPH from AD.
